# [Retracted] ABT-737, a Bcl-2 family inhibitor, has a synergistic effect with apoptosis by inducing urothelial carcinoma cell necroptosis

**DOI:** 10.3892/mmr.2025.13705

**Published:** 2025-10-06

**Authors:** Rui Cheng, Xiaolong Liu, Zheng Wang, Kunlong Tang

Mol Med Rep 23: 412, 2021; DOI: 10.3892/mmr.2021.12051

Following the publication of the above paper, it was drawn to the Editor's attention by a concerned reader that certain of the western blot data shown in Fig. 2A on p. 5 had already appeared in an article written by different authors at largely different research institutes that was published previously in the journal *Oncotarget*.

Owing to the fact that the contentious data in the above article had already been published prior to its submission to *Molecular Medicine Reports*, the Editor has decided that this paper should be retracted from the Journal. The authors were asked for an explanation to account for these concerns, but the Editorial Office did not receive a reply. The Editor apologizes to the readership for any inconvenience caused.

